# Mechanisms of Neuroblastoma Cell Growth Inhibition by CARP-1 Functional Mimetics

**DOI:** 10.1371/journal.pone.0102567

**Published:** 2014-07-17

**Authors:** Magesh Muthu, Vino T. Cheriyan, Sara Munie, Edi Levi, John Frank, Abdelkader E. Ashour, Mandip Singh, Arun K. Rishi

**Affiliations:** 1 John D. Dingell VA Medical Center, Wayne State University, Detroit, Michigan, United States of America; 2 Karmanos Cancer Institute, Wayne State University, Detroit, Michigan, United States of America; 3 Oncology Department, Wayne State University, Detroit, Michigan, United States of America; 4 Pathology Department, Wayne State University, Detroit, Michigan, United States of America; 5 Department of Pharmacology and Toxicology, College of Pharmacy, King Saud University, Riyadh, Kingdom of Saudi Arabia; 6 College of Pharmacy and Pharmaceutical Sciences, Florida A&M University, Tallahassee, Florida, United States of America; University of Nebraska Medical Center, United States of America

## Abstract

Neuroblastomas (NBs) are a clinically heterogeneous group of extra cranial pediatric tumors. Patients with high-risk, metastatic NBs have a long-term survival rate of below 40%, and are often resistant to current therapeutic modalities. Due to toxic side effects associated with radiation and chemotherapies, development of new agents is warranted to overcome resistance and effectively treat this disease in clinic. CARP-1 functional mimetics (CFMs) are an emerging class of small molecule compounds that inhibit growth of diverse cancer cell types. Here we investigated NB inhibitory potential of CFMs and the molecular mechanisms involved. CFM-1, -4, and -5 inhibited NB cell growth, in vitro, independent of their p53 and MYCN status. CFM-4 and -5 induced apoptosis in NB cells in part by activating pro-apoptotic stress-activated kinases (SAPKs) p38 and JNK, stimulating CARP-1 expression and cleavage of PARP1, while promoting loss of the oncogenes C and N-myc as well as mitotic cyclin B1. Treatments of NB cells with CFM-4 or -5 also resulted in loss of Inhibitory κB (IκB) α and β proteins. Micro-RNA profiling revealed upregulation of XIAP-targeting miR513a-3p in CFM-4-treated NB, mesothelioma, and breast cancer cells. Moreover, exposure of NB and breast cancer cells to CFM-4 or -5 resulted in diminished expression of anti-apoptotic XIAP1, cIAP1, and Survivin proteins. Expression of anti-miR513a-5p or miR513a-5p mimic, however, interfered with or enhanced, respectively, the breast cancer cell growth inhibition by CFM-4. CFMs also impacted biological properties of the NB cells by blocking their abilities to migrate, form colonies in suspension, and invade through the matrix-coated membranes. Our studies indicate anti-NB properties of CFM-4 and 5, and suggest that these CFMs and/or their future analogs have potential as anti-NB agents.

## Introduction

Neuroblastoma (NB) is the most common malignant extra cranial solid tumor of children, and account for 8–10% of pediatric cancers [1]. Higher stage of disease, age of >18 months, MYCN amplification, and unfavorable histology are indicators of poor prognosis [1], [2]. The current treatment regimens include high-dose chemotherapy with autologous stem cell transplantation, radiation and surgery. In the high-risk metastatic NBs, the long-term survival rates are <40% [3], [4]. However, NB frequently relapses with resistant disease due in part to selection of drug-resistant cells during treatment [5]. Therefore, new therapeutic strategies are needed to overcome drug resistance and improve anti-neuroblastoma treatment outcomes.

Cell cycle and apoptosis regulator 1 (CCAR1/CARP-1) is a peri-nuclear phospho-protein, that regulates cell growth and apoptosis signaling in a variety of cancer cells [6]–[8]. CARP-1 functions as a key transcriptional co-activator of steroid family of nuclear receptors and tumor suppressor p53 in regulating Adriamycin (ADR)-dependent DNA damage-induced apoptosis. Increased CARP-1 expression also occurs during cell cycle arrest and apoptosis following withdrawal of the serum growth factors [6]–[8]. Recent studies revealed that CARP-1 phosphorylation plays a significant role in mediating apoptosis. For example, apoptosis stimulation following blockage of EGFRs involves CARP-1 phosphorylation at tyrosine^192^, activation of p38 MAPK and caspase-9. Pharmacologic inhibition of protein kinase A (PKA) results in CARP-1 threonine^667^ phosphorylation, abrogation of c-Myc transcription and inhibition of human breast cancer cell growth [8], [9]. Depletion of CARP-1, on the other hand, resulted in resistance to apoptosis with ADR or EGFR tyrosine kinase inhibitors [6].

Our recent studies demonstrated that CARP-1 also functions as a co-activator of cell cycle regulatory anaphase promoting complex/cyclosome (APC/C) E3 ligase [10]. APC/C is a multi-subunit ubiquitin E3 ligase protein that plays a distinct role in cell cycle transitions [11], [12]. Previous studies showed that misregulation of APC/C and its substrates correlates with tumor progression [13]. We identified a novel class of small molecule inhibitors (SMIs) of CARP-1 binding with APC/C subunit APC2. These compounds, termed CARP-1 functional mimetics (CFMs), inhibit cell growth by inducing apoptosis in various cancer types [10], [14], [15]. Here we provide evidence that CFMs are novel and potent inhibitors of NB cell growth.

## Materials and Methods

### Cells and reagents

Four human NB cell lines (SK-N-AS, SK-N-DZ, SK-N-BE(2), and SK-N-SH) were purchased from ATCC, and were kindly provided by Dr. Yubin Ge, Karmanos Cancer Institute, Wayne State University, Detroit, MI. The NB cells were routinely cultured either in the RPMI-1640 (SK-N-BE(2) and SK-N-SH) or in DMEM (SK-N-AS, SK-N-DZ) medium that was supplemented with 10% FBS, 100 units/ml of penicillin, and 100 µg/ml of streptomycin. Cells were maintained at 37°C and 5% CO_2_ [16]. Human breast cancer (HBC) MDA-MB-468 and MDA-MB-231 cells (that lack estrogen receptor and have mutant p53) were also purchased from ATCC, and routinely cultured in our laboratory essentially as described [6]. MDA-MB-468 subline (AS clone 9) expressing reduced CARP-1 following stable expression of CARP-1 antisense were generated and characterized as detailed before [6], while malignant pleural mesothelioma (MPM) H2373 cells were cultured as described previously [14].

DMEM, RPMI-1640 medium, penicillin and streptomycin were purchased from Invitrogen Co. (Carlsbad, CA). CFM-1, -4 and -5 were obtained from ChemDiv, San Diego, and Ryan Scientific, Inc., Mt. Pleasant, SC, and were dissolved in dimethyl sulfoxide (DMSO) at a stock concentration of 10, 50, and 50 mM, respectively, and stored at −20°C. FBS was purchased from Denville Scientific Inc. (Metuchen, NJ), and DMSO was purchased from Fischer Scientific (Fair Lawn, NJ). Anti-β-actin mouse monoclonal antibody, and 3-4, 5-dimethyltiazol-2-yl-2.5-diphenyl-tetrazolium bromide (MTT) were purchased from Sigma-Aldrich (St. Louis, MO). The monoclonal antibodies for ABIN2, the polyclonal antibodies for TIMP2 (goat polyclonal), DR4 and DR5 were obtained from Santa Cruz Biotech, Santa Cruz, CA. The mouse monoclonal antibody for α-tubulin was obtained from Calbiochem (Billerica, MA). Anti-cyclin B1, anti phospho-JNK (Threonine183/Tyrosine185) G9 mouse monoclonal antibodies, anti-JNK (56G8), anti-XIAP (3B6), anti-Survivin (71G4B7) rabbit monoclonal antibodies, and rabbit polyclonal antibodies for PARP, phospho and total p38α/β SAPK, ABIN1, IκBα, IκBβ, and c-IAP1 were obtained from Cell Signaling Technology (Beverly, MA). Rabbit polyclonal antibody for MYCN was obtained from Abcam (Cambridge, MA). Generation and characterization of the anti-CARP-1/CCAR1 rabbit polyclonal antibodies have been described elsewhere [6]. Enhanced Chemiluminescence Reagent was purchased from Amersham Biosciences (Piscataway, NJ) and the Protein Assay Kit was purchased from Bio-Rad Laboratories (Hercules, CA). The NF-κB-TATA-Luc plasmid that contains 5x NF-κB consensus cis sequences/enhancer positioned upstream of the TATA elements that drive firefly luciferase reporter expression, and the plasmid for expression of Renilla luciferase (pTK/Renilla Luc used as internal control for transfections) were purchased from Stratagene, Inc. (LaJolla, CA) and Promega, Inc (Madison, WI), respectively.

### Cell cycle analysis, MTT, apoptosis and Western blot assays

The cell cycle distribution was analyzed by flow cytometry. NB cells (1×10^6^) were untreated or treated with respective CFM, and harvested and washed in PBS. The cells were then fixed in 70% alcohol for 30 min at 4°C. The cells were subsequently washed thrice in cold PBS, and suspended in 1 ml of PBS containing 50 µg of propidium iodide and 100 µg of RNAseA for 30 min at 37°C. The cells were then analyzed for their DNA content by FACSCalibur (Becton-Dickinson, Mountain View, CA).

In vitro inhibition of cell growth was assessed by MTT (3-[4, 5-dimethyltiazol-2-yl]-2.5-diphenyl tetrazolium bromide) reagent. Cells (5×10^3^) were seeded in a 96-well culture plate and subsequently treated with respective CFMs and Adriamycin (ADR) at different concentrations as mentioned. Control cells were treated with 0.1% DMSO in culture medium. After treatment, the cells were incubated with 1 mg/ml of MTT reagent at 37°C for 2–4 hours and then MTT was removed and 50 µL of DMSO was added, followed by colorimetric analysis using a multi-label plate reader at 560 nm (Victor3; PerkinElmer, Wellesley, MA, USA).

Apoptosis levels were determined by staining for fragmented DNA utilizing terminal deoxynucleotidyl transferase-mediated nick end labeling (TUNEL) assay. TUNEL kits were purchased from Roche Diagnostics, (Indianapolis, IN). For TUNEL labeling, the cells were either untreated or treated with 5 or 10 µM of CFM-1, -4, -5 for 12 h. The slides were rinsed to remove the media, and the cells were fixed for staining using a 1∶250 dilution of anti-CARP-1 (α2), anti-PARP1, anti-MYCN or anti-c-Myc antibodies, or 1∶500 dilution of the anti-phospho-p38 antibody respectively. The fixed and labeled cells were photographed essentially as detailed in our previously described methods [17].

For protein expression analysis, Western blot (WB) experiments were done according to the standard procedures. The cells were either untreated or treated with CFMs and ADR, harvested and lysed in cell lysis (10X) buffer (#9803; cell signaling) containing 0.1% of protease and phosphatase inhibitor cocktail (Sigma) for 20 min at 4°C. The lysates were centrifuged at 14,000 rpm at 4°C for 15–20 min to remove debris. Protein concentrations of whole cell lysates were determined using the Protein Assay Kit. Supernatant proteins, 50–100 µg from each sample, were separated by SDS-polyacrylamide gel electrophoresis (SDS-PAGE) and transferred to polyvinylidene difluoride (PVDF) membrane (Bio-rad, Hercules, CA) by standard procedures. The membranes were hybridized with primary antibodies followed by incubation with appropriate secondary antibodies. The antibody-bound proteins were visualized by treatment with the chemiluminescence detection reagent according to manufacturer's instructions, followed by exposure to X-ray film (Denville Scientific Inc.). The same membranes were re-probed with the anti-β-actin or anti-α-tubulin antibody, which was used as an internal control for protein loading.

### Luciferase assays

Cells were plated either in a 12-well plate or in a 24-well plate at a density of 3×10^5^ cells/ml and then transfected with pTK/Renilla Luc in combination with NF-κB-TATA-Luc essentially following previously detailed methods [6], [8]. After 5 h incubation with plasmid DNAs, FBS was added to the transfection media and cells were allowed to grow for at least 18 h. Cells were left untreated or treated with TNFα, ADR, or CFM-4. The cells were then harvested, lysed, and Renilla and firefly luciferase activities were measured using dual luciferase assay kit (Promega) essentially following vendors' guidelines.

### Cell migration, invasion and Clonogenic assays

The NB cells migration in the presence of CFMs was measured by the “scratch/wound healing” assay. Cells were seeded in a 6-well plate (∼10,000 cells/well), and when attached, a scratch was created in the cell monolayer using sterile pipette tip. The cells were then allowed to continue growing in the absence (Control) or presence of 3 µM dose of each of the CFMs for additional 72–96 h. The cells were photographed at the beginning and at regular intervals during the treatment period, and the images from control cells were compared with the treated cells to determine the migration of the cells essentially as described before [18]. The photomicrographs of the cells were recorded under different magnifications utilizing Zeiss microscope with attached 35 mm camera.

#### Invasion assay

An in vitro assay using matrigel was utilized for invasion assay. Since the metastatic tumor cells often produce proteases that degrade the extracellular matrix (ECM) to facilitate their migration through stroma, the in vitro assay using Matrigel is considered to be the most reliable, reproducible, and representative of in vivo invasion by the cancer cells. In this assay, cancer cells are placed in the upper chamber that is separated from the lower chamber by a porous membrane coated with Matrigel [17], [19]. A cell invasion Boyden chamber assay kit (Chemicon International, CA) was utilized to measure invasion properties of the NB cells in the absence or presence of CFMs. Briefly, 300 µl of pre-warmed serum free medium was used to hydrate the ECM layer of each chamber for 15–30 minutes at room temperature. Approximately 2–2.5×10^5^ NB cells were then seeded in the upper chamber in a serum-free medium without or with respective CFMs. Since 10 µM dose of CFMs elicited extensive cell death in NB cells, a slightly lower dose of 7 µM for each CFM was utilized over a 24 h treatment period for these assays. The lower chamber was supplied with medium containing 10% FBS that served as chemo-attractant to stimulate migration. After an interval, tumor cells present on the lower side of the membrane in the lower chamber were stained, and photographed as above. In addition, the stained cells from the lower side of membrane of some wells were dissociated, lysed in a buffer, followed by quantitation using a fluorescence plate reader with 480/520 nm filter set. The measurements were then plotted as columns in histogram.

#### Clonogenic assay

A soft-agar sandwitch assay was performed. Cells were sandwiched between 0.6% and 0.3% agarose in DMEM medium containing 5% FBS in a six-well chamber (500 cells/chamber), and treated with buffer (Control), or respective CFM (10 µM) for 9 days at 37°C humidified CO_2_ incubator. The colonies from multiple random fields were counted, compared to control and photographed essentially as described before [14], [15], [17].

### Detection of MMP & TIMP expression in NB cells

SK-N-SH cells were either untreated, separately treated with CFM-4 or CFM-5. After treatment, the cells were homogenized in RIPA buffer (500 µl of lysis buffer per 1×10^6^ cells), followed by centrifugation of lysates at 10,000×g for 5 min. The protein concentration in the respective supernatant was determined by using Bicinchoninic acid assay, and the lysates were stored at -80°C until further use. MMP and TIMP activation in each lysate was measured using the Quantibody reverse phase human MMP array kit according to manufacturer's instructions (RayBiotech, Norcross, GA). Fluorescence images were detected using a GenePix 4100A Scanner, and data was analyzed using the QAH-MMP-1 GAL software based on the instruction provided by the array manufacturer.

### MiRNA profiling

The SK-N-SH NB and H2373 MPM cells were either untreated or treated with 20 µM dose of CFM-4 for 3, 6, 12, and 24 h periods in serum-free medium. In addition, MDA-MB-468 HBC cells were separately treated with 20 µM dose of CFM-4 for 1, 6, and 12 h periods in a serum-free medium. At the end of treatments, the untreated and treated cells were harvested in 1 ml of Trizol reagent (InVitrogen) and total RNAs were extracted according to the manufacturer's protocols. Determination of RNA quality, labelling, hybridization with miRNA arrays, scanning and image analysis, and data analysis were custom performed by Exiqon Inc., Denmark. Briefly, the quality of the RNAs was first verified by an Agilent 2100 Bioanalyzer profile, and 750 ng of each of the RNA was labeled with Hy3 and Hy5 fluorescent label, respectively, using the miRCURY LNA microRNA Hi-Power Labeling Kit (Hy3/Hy5; Exiqon, Denmark) following the procedure described by the manufacturer. The Hy3-labeled samples and a Hy5-labeled reference RNA sample were then mixed pair-wise and hybridized to the miRCURY LNA microRNA Array 7th gen (Exiqon, Denmark), which contains capture probes targeting all microRNAs for human, mouse or rat that are currently registered in the miRBASE 18.0. The hybridization was performed utilizing a Tecan HS4800 hybridization station (Tecan, Austria). After hybridization the microarray slides were scanned and stored in an ozone free environment (ozone level below 2.0 ppb) in order to prevent potential bleaching of the fluorescent dyes. The miRCURY LNA microRNA Array slides were scanned using the Agilent G2565BA Microarray Scanner System (Agilent Technologies, Inc., USA) and the image analysis was carried out using the ImaGene 9.0 software (BioDiscovery, Inc., USA). The quantified signals were background corrected (Normexp with offset value 10) as described [20], and normalized using quantile normalization method to minimize the intensity-dependent differences between the samples. The differentially expressed miRNAs with absolute value of log fold change larger than 1 compared to control were selected for further analysis and validation.

### Anti-miR-513a-5p and miR-513a-5p mimic transfection

Anti-miR-513a-5p, miR-513a-5p mimics and negative control were purchased from Bioneer (Alameda, CA). 50 nM of Anti-miR-513a-5p, miR-513a-5p mimic or scrambled negative control were transfected using Lipofectamine RNAiMAX (Invitrogen) in serum free medium following manufacturer's instructions. After 96 h incubation with miRs, the cells were either lysed, and protein extracts were analyzed by WB for expression of miR-513a-5p target XIAP protein, or cells were treated with CFM-4 and their viabilities were determined by MTT assay as above.

### Statistical analysis

In some instances, statistical analysis was performed using unpaired Student's t-test. A p-value less than 0.05 between treatment groups was considered significantly different.

## Results

### NB cell growth suppression by CFMs involves stimulation of apoptosis

**Figure 1:**
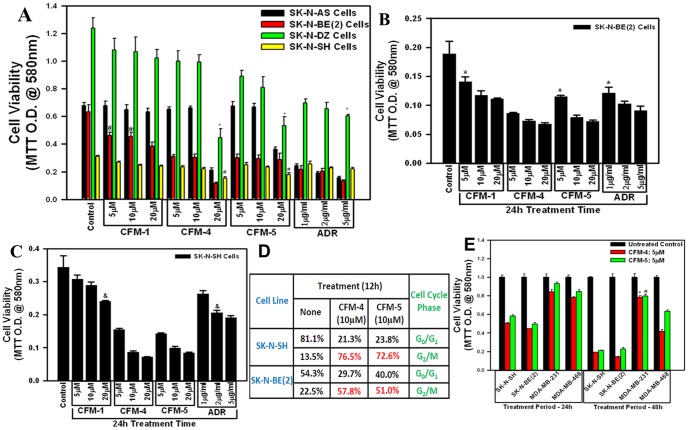
CFMs reduce viabilities of the NB cells. Cells were treated with vehicle (Untreated Control), indicated doses of ADR, CFM-1, CFM-4, or CFM-5, 12 h (A) or 24 h (B, C, E). Determination of viable/live cells was carried out by MTT assay. In panel D, NB cells were treated with indicated dose and time of CFM-4 or CFM-5, labeled with propidium iodide, and sorted by flow cytometry. The table represents % cell numbers in respective cell cycle phase. The data in the histograms represent means of three independent experiments; bars, S.E. @, #, * and &, p = <0.05 relative to respective untreated Controls.

Our previous studies have indicated cancer cell growth inhibitory properties of CFMs in particular CFM-4 and CFM-5 [10]. In this study, we utilized a number of NB cells (SK-N-SH, SK-N-BE(2), SK-N-AS, SK-N-DZ) to investigate their growth inhibition by CFMs. In the first instance, we treated the NB cells with 5, 10, or 20 µM doses of each CFM or 1, 2, or 5 µg/ml dose of ADR for a period of 12 h. As shown in figure 1A, CFM-4 and -5 inhibited viability of all the four cell lines in a dose dependent manner. All the NB cells were inhibited to similar degree by different doses of ADR with the exception of the SK-N-DZ cells that were relatively less sensitive to inhibition by ADR. The growth of the NB cells, with the exception of SK-N-BE(2) cells, was not affected by treatments with CFM-1 (Figure 1A). SK-N-SH and SK-N-AS cells harbor single copy of MYCN and WT p53; while the SK-N-BE(2) have MYCN amplification and mutated p53 [21]–[23]. Attenuation of different NB cell growth by CFM-4, CFM-5, or ADR would suggest for their growth suppression independent of the involvement of MYCN and/or p53 signaling. Although ADR is currently utilized in clinic to treat NB, and the tumors initially respond to ADR therapy, the emergence of ADR resistance remains a significant and unresolved concern [21]. Since ADR inhibited growth of SK-N-BE(2) cells while SK-N-SH cells were either unaffected or moderately affected by ADR treatments, we selected SK-N-SH and SK-N-BE(2) in a proof-of-concept study to further test the efficacy of CFM-4 and CFM-5 compounds. Both the NB cells were separately treated with different doses of CFMs or ADR as in figure 1A except that the treatment period was extended to 24 h. A 20 µM dose of CFM-1 as well as 1 µg/ml dose of ADR caused ∼30–33%loss of viability of both the NB cell lines (Figure 1B, C). The 20 µM dose of CFM-4 or CFM-5 over a 24 h treatment period induced ∼70–80% loss of viability of both the NB cells (Figure1B, C). Treatments of both the NB cells with 10 or 20 µM dose of either CFM-4 or CFM-5 elicited consistently and significantly higher loss of cell viabilities when compared with all the doses of ADR tested (Figure 1B, C). Since a 5 µg/ml concentration of ADR corresponds to ≈9.2 µM dose, and the data in figures 1B and C show a greater inhibition of the NB cell growth by 10 µM dose of each of the CFM-4 or CFM-5, it is likely that either of the CFM is superior inhibitor of the NB growth in comparison with ADR. Because CFM-4 and CFM-5 bind with CARP-1 and interfere with APC/C E3 ubiquitin ligase functions to regulate cell cycle [10], we determined whether these compounds interfere with NB cell cycle progression. Flow cytometric analysis revealed that, like the HBC cells, both the CFMs caused accumulation of NB cells in G2M phase (Figure 1D). An MTT-based analysis of the NB and HBC cells treated with 5 µM dose of CFM-4 or CFM-5 over a period of 48 h further revealed a moderate to minimal loss of viability of the HBC cells while CFM-4 or CFM-5 treatments resulted in ∼70–80% reduction of NB cells viabilities (Figure 1E). Collectively, the data in figure 1 suggest that CFM-4 and CFM-5 are effective and superior inhibitors of NB cell growth, and NB cells are highly sensitive to inhibition by CFM-4 or CFM-5 when compared with the HBC cells.

**Figure 2:**
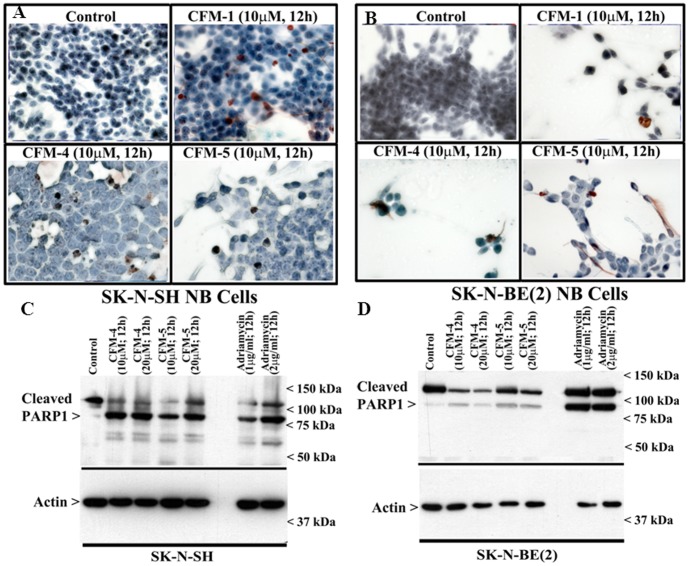
CFMs induce apoptosis in NB cells. (A, B) Indicated NB cells were either untreated (Control) or treated with 10 µM dose of respective CFMs for 12 h. Staining of the cells was performed using TUNEL assay as detailed in Methods. Dark brown staining represents fragmented cell nuclei. (C, D) Cells were either untreated (Control) or treated with indicated agents for noted time and dose, and levels of cleaved PARP and actin proteins were determined by Western blotting.

We next determined whether CFMs promoted apoptosis to inhibit NB cell growth. Based on our MTT analysis, where treatments with 10 or 20 µM doses of CFM-4 or -5 elicited significant ∼50–80% loss of viability over a period of 24 h in both the NB cells, while CFM-1 was found to be modestly active, we chose to utilize 10 and 20 µM doses of CFM-4 and CFM-5 for further experiments to explore the molecular mechanisms of cell growth suppression and apoptosis stimulation. For determination of apoptosis, we performed DNA fragmentation-based TUNEL assay as detailed in methods. Treatments of SK-N-SH or SK-N-BE(2) NB cells with CFMs resulted in increased number of TUNEL-positive cells (Figure 2A, B). Further, WB analysis also revealed increased expression of cleaved PARP-1 (poly(ADP-ribose) polymerase-1), a marker of apoptosis, following 12 h treatment of NB cells with the 10 and 20 µM dose of CFM-4 or CFM-5 (Figure 2C, D). Consistent with earlier findings that demonstrated involvement of PARP-1 cleavage during ADR-induced apoptosis [24], treatment of NB cells with 1 and 2 µg/ml doses of ADR for 12 h also resulted in cleavage of PARP-1. These data suggest that CFMs, particularly CFM-4 and CFM-5, inhibit NB cell growth in part by inducing apoptosis.

### CFMs promote apoptosis in NB cells by inducing phosphorylation of p38 MAP kinase, c-Jun N-terminal kinase (JNK) and stimulating expression of CCAR-1/CARP-1

**Figure 3:**
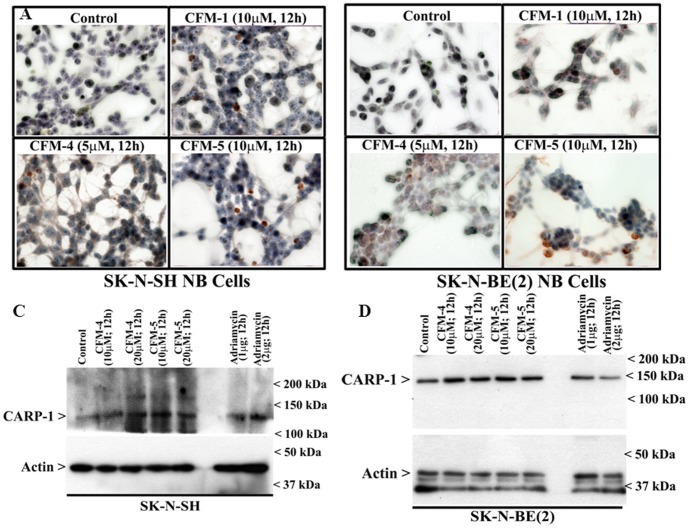
CFMs upregulate pro-apoptotic CARP-1 levels. (A, B) Indicated NB cells were either untreated (Control), treated with respective CFMs as in figure 2. Staining of the cells was performed using anti-CARP-1 (α2) antibody as detailed in Methods. Presence of CARP-1 is indicated by intense brown staining in the nuclei and cytosol of the treated cells. (C, D) Cells were either untreated (Control) or treated with different agents for indicated dose and time, and cell lysates were analyzed by Western blotting for levels of CARP-1 and actin proteins as in Methods.

CARP-1 has previously been shown to function as a co-activator of p53 tumor suppressor functions following treatments of cells with ADR [6], [7]. Moreover, knock-down of CARP-1 resulted in elevated levels of topoisomerase IIα [6] and also interfered with HBC cell growth inhibition by CFM-4 [10]. To determine whether NB cell growth suppression and apoptosis induction involved CARP-1 expression, we first analyzed CARP-1 levels in NB cells that were treated with CFM-4 or CFM-5 by performing immuno-cytochemical staining as noted in methods. As shown in figure 3A, B, immuno-cytochemical analysis revealed elevated CARP-1 levels in CFM-treated NB cells. Additional WB analysis of the NB cells that were treated with different doses of CFM-4, CFM-5, or ADR, showed increased CARP-1 levels when compared with CARP-1 levels in their respective, untreated controls (Figure 3C, D).

**Figure 4:**
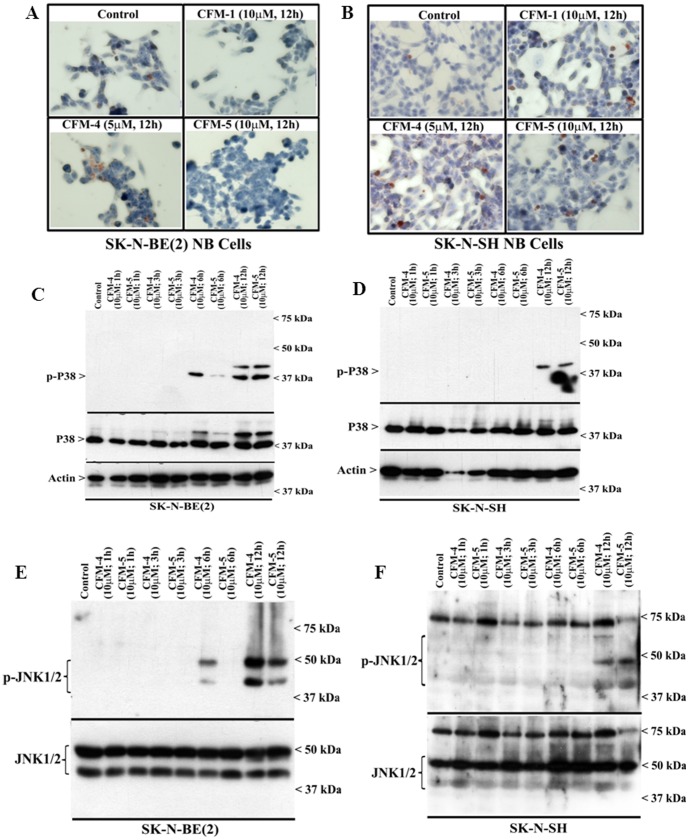
CFMs activate pro-apoptotic SAPKs in NB cells. (A, B) Indicated cells were either untreated (Control) or treated with respective CFMs as in figure 2A. Staining of the cells was performed using anti-phospho-p38 antibody as detailed in Methods. Presence of p38 is indicated by intense brown staining in the nuclei and cytosol of the treated cells. NB cells were either untreated (Control) or treated with indicated CFMs for noted time and dose, and levels of phosphorylated p38 (noted as p-p38), and total p38 proteins (C, D) or phosphorylated JNK (noted as p-JNK1/2), and total JNK proteins (E, F) were determined by Western blotting essentially as in figure 2.

A number of earlier reports have demonstrated activation of p38 and/or JNK SAPKs in transducing apoptosis signaling in NB cells [24], [25]. To determine the extent SAPKs are also activated by CFMs, we conducted immuno-cytochemical staining of the CFM-treated and untreated NB cells for presence of phosphorylated (activated) p38α/β. As shown in figure 4A, B, treatments with CFMs resulted in elevated staining for phosphorylated p38α/β. Further WB analyses revealed increased phosphorylation of p38α/β as well as JNK1/2 in the CFM-4 or CFM-5-treated NB cells in a time dependent manner (Figure 4C–F). These data suggest that NB cell growth inhibition by CFMs involves activation of SAPKs, and are in agreement with our previous studies demonstrating stimulation of CARP-1 and activation of SAPKs by CFMs in HBC, medulloblastoma (MB) and MPM cells [14], [15].

### CFMs suppress MYCN and c-Myc expression while activating NF-κB signaling

**Figure 5:**
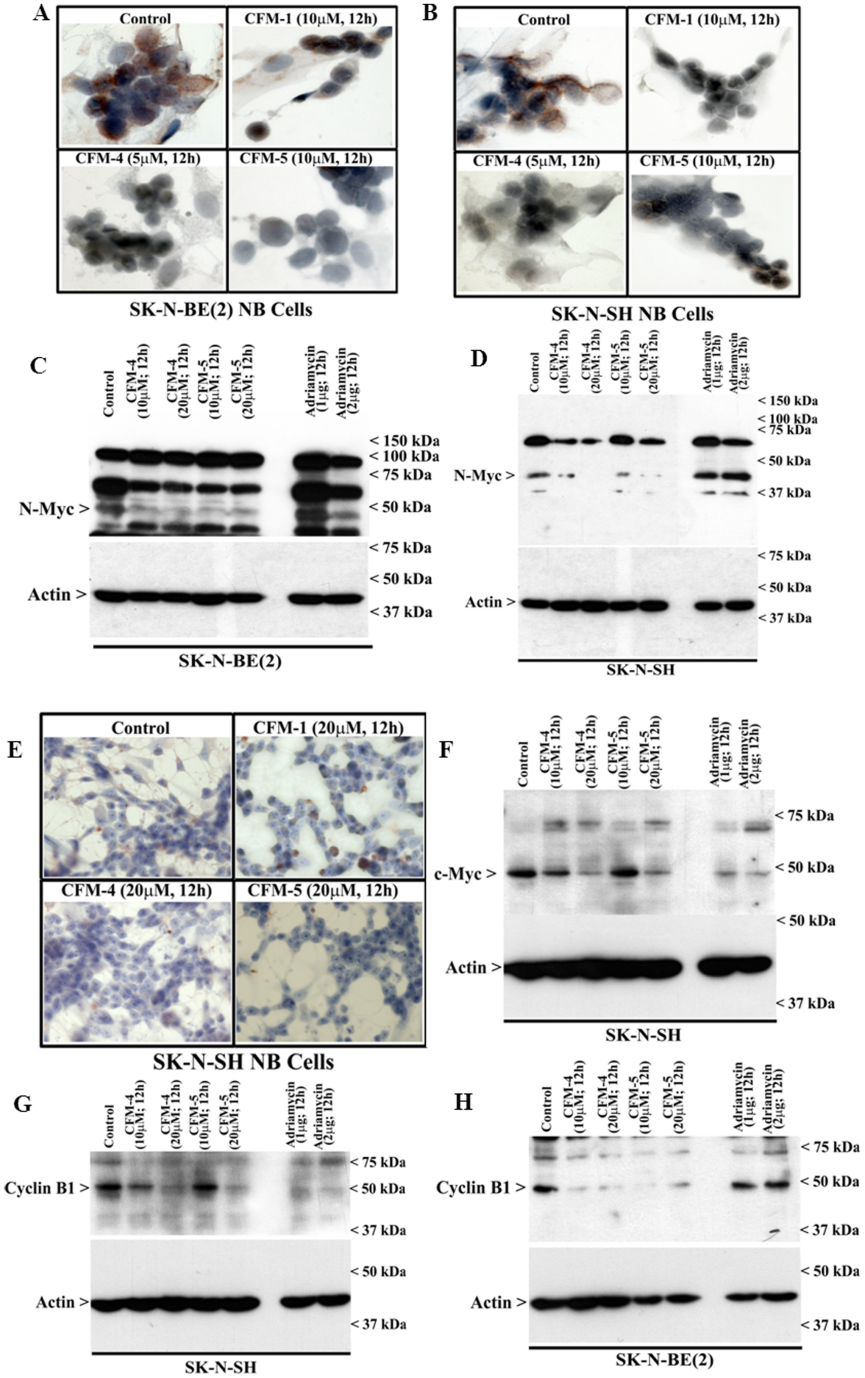
CFMs suppress expression of oncogenes N and c-myc. (A, B, E) Cells were either untreated (Control), treated with indicated time and dose of respective CFMs, and followed by staining of cells using anti-N-myc (A, B) or c-myc (E) antibody as detailed in Methods. Presence of N-myc or c-myc proteins is indicated by intense brown staining in the nuclei of the untreated cells. (C, D, F-H) Cells were either untreated (Control) or treated with indicated agents for noted time and dose, and cell lysates were analyzed by Western blotting for levels of N-myc (C, D), c-myc (F), or cyclin B1 (panels G and H) and actin proteins as in Methods.

MYCN amplification plays a significant role in development of NB and is considered to be a key therapeutic target for NB [26]. We asked whether CFMs target MYCN in NB cells. Immuno-cytochemical analysis revealed that treatment of SK-N-SH and SK-N-BE(2) cells with 5 or 10 µM dose of respective CFMs resulted in diminished staining for MYCN (Figure 5A, B). The decline in MYCN levels in CFM-treated NB cells was further confirmed by WB analysis. Both the CFMs caused a noticeable decline in MYCN levels in SK-N-BE(2) cells (Figure 5C), a more pronounced loss of MYCN was however noticed in CFM-treated SK-N-SH cells (Figure 5D). Of note here is that ADR treatments failed to provoke any loss of MYCN in either of the NB cells (Figure 5C, D). Our previous studies noted that while stimulating CARP-1 expression and activation of SAPKs, CFM-4 also caused loss of mitotic cell cycle regulator cyclin-B1, cell growth and migration regulatory small GTP-binding protein p21Rac1, and oncogene c-Myc [10]. Consistent with these observations, our current immunocytochemical analysis show reduced levels of c-Myc in CFM-5-treated cells albeit a moderate loss of c-Myc staining was also noted in SK-N-SH cells that were treated with CFM-1 or CFM-4 (Figure 5E). WB analysis further show that although a 10 µM dose of CFM-4 or CFM-5 elicited a moderate loss of c-Myc expression, exposure of SK-N-SH NB cells to ADR or a 20 µM dose of each of the CFMs however caused a more pronounced loss of c-Myc expression (Figure 5F). Moreover, exposure to CFM-4 also resulted in a pronounced loss of cyclin B1 levels in both the NB cells, and a moderate and robust loss of cyclin B1 occurred in CFM-5-treated SK-N-SH and SK-N-BE(2) cells, respectively. ADR, on the other hand, was able to target cyclin B1 in SK-N-SH, but not in SK-N-BE(2) NB cells (Figure 5G, H). These data collectively suggest that CFM-4 and CFM-5 signaling likely overlap with ADR in down-regulating key cell proliferation and survival regulating genes to inhibit growth of the NB cells, and both the compounds are superior in suppressing MYCN expression when compared with ADR.

**Figure 6:**
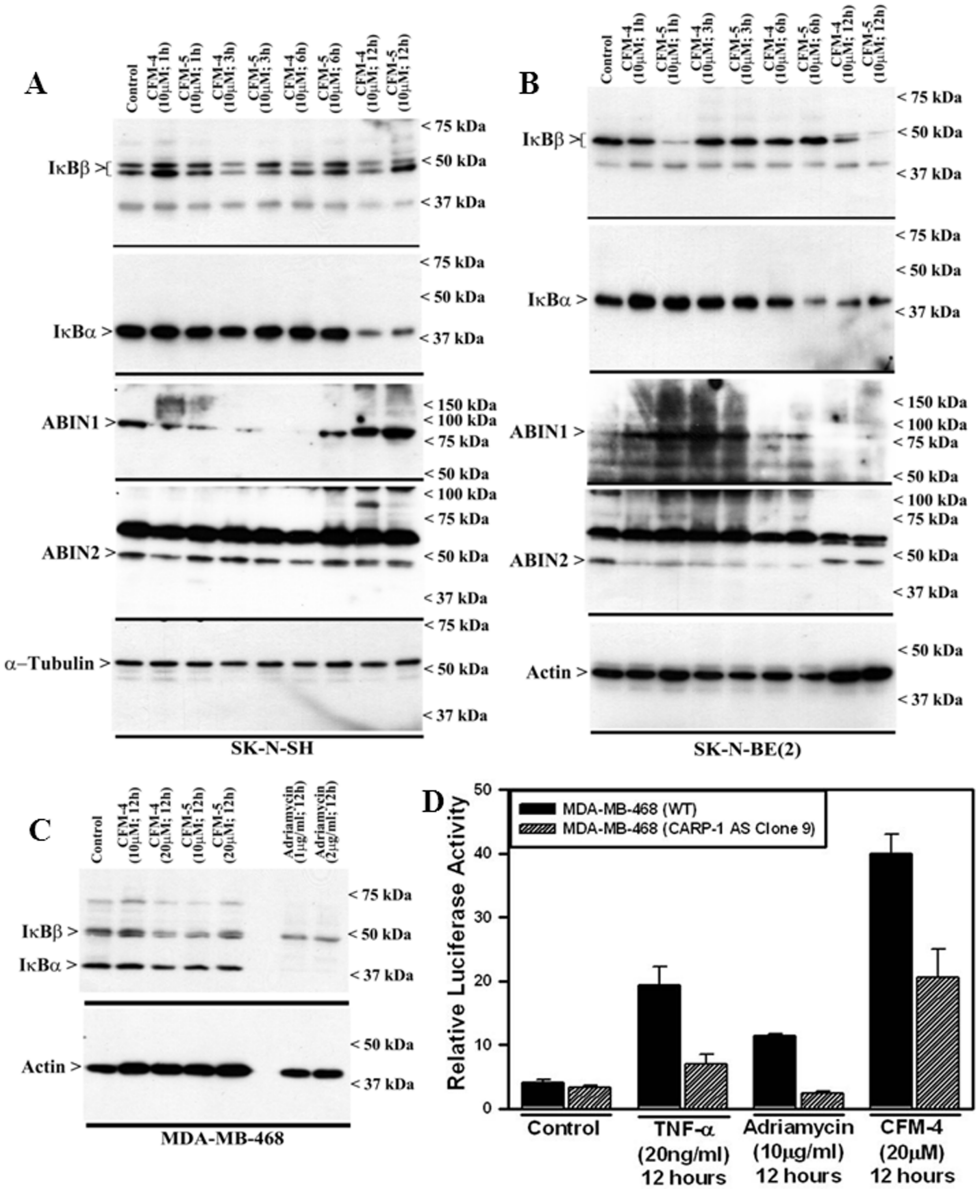
CFM-4 activates NF-κB signaling. SK-N-SH (A) and SK-N-BE(2) (B) NB cells were either untreated (Control) or treated with noted CFMs for indicated dose and time, and cell lysates were analyzed by Western blotting for levels of ABIN1, ABIN2, IκBα, IκBβ, α-tubulin and actin proteins as indicated in Methods. HBC cells were either untreated (Control) or treated with indicated agents for noted time and dose, and levels of IκBα, IκBβ (C), and actin proteins were determined by WB essentially as in figure 2. (D) CFM-4 induces transcriptional activation of NF-κB. Cells were transfected with NF-κB-TATA-Luc and TK-Renilla-Luc reporter plasmids followed by their treatments with various agents for noted dose and time. Cell lysates were utilized to determine firefly and Renilla luciferase (internal control) activities as in methods. Columns in the histogram represent relative luciferase activities from two independent experiments; bars, S.E.

NF-κB is a protein complex that regulates transcription of DNA, and many physiological processes, including cell death and inflammation. Dysregulation of NF-κB is often encountered in many human diseases, including cancer [27]. Since IκBα, IκBβ, ABIN-1 and ABIN-2 are known negative regulators of NF-κB [28]–[30], and in light of our previous studies demonstrating diminished expression of these negative regulators of NF-κB signaling in MPM and MB cells that were exposed to CFM-4 [14], [15], here we tested whether treatments of NB cells with CFM-4 or CFM-5 also reduced expression of IκBα, IκBβ, ABIN1 and ABIN2 proteins. Both SK-N-SH and SK-N-BE(2) NB cells that were exposed to 10 µM dose of CFM-4 or -5 over a period of 12 h had reduced levels of IκBα and IκBβ proteins when compared with their untreated controls (Figure 6A, B). Treatments with either CFMs failed to significantly alter expression of ABIN2 in SK-N-SH cells while biphasic alteration in ABIN2 expression was noted in SK-N-BE(2) cell that were treated with either CFMs (Figure 6A, B). Both the CFMs caused reduced levels of ABIN2 in SK-N-BE(2) cells over the periods of 1, 3, and 6 h, while minimally affecting its levels over a 12 h treatment period (Figure 6B). Likewise a similar biphasic regulation of ABIN1 was also noted in CFM-treated NB cells. In SK-N-SH cells, treatments with CFMs over 1, 3, or 6 h periods caused diminished levels of ABIN1 while a 12 h treatment period elicited a minimal effect on ABIN1 expression (Figure 6A). On the other hand, in SK-N-BE(2) cells, CFM treatments over 1 and 3 h periods caused robust increase in ABIN1 expression, while no such increase was evident when these cells were treated with either CFM over the 6 or 12 h periods (Figure 6B). These data indicate that by regulating cellular levels of ABINs, CFMs likely modulate canonical NF-κB signaling in a manner dependent on cell-type and treatment durations. However, a consistent depletion of IκBs by either of the CFMs in both the NB cells over a 12 h treatment period would argue for activation of NF-κB signaling by these compounds.

Our previous studies with MB and MPM cells [14], [15] together with our current studies with NB cells suggest that CFMs likely activate NF-κB signaling. Since CFMs were previously found to inhibit growth of the HBC cells [10] we next determined whether and to the extent CFM-4 also activated NF-κB signaling in HBC cells. MDA-MB-468 HBC cells were either untreated, treated with CFM-4, CFM-5, or ADR followed by WB analysis of their lysates for levels of IκBα and IκBβ proteins. Consistent with diminished levels of IκBα and IκBβ proteins in the NB cells that were treated with CFM-4 or CFM-5 compounds, exposure of the HBC cells to CFM-4, CFM-5, or ADR also caused reduced levels of IκBα and IκBβ proteins (Figure 6E). Additional WB analysis of the cell lysates derived from the NB and HBC cells that were treated with CFM compounds failed to reveal loss of expression of the NF-κB subunits p65 RelA and p50 (not shown). The reduced levels of IκBα and IκBβ proteins without affecting the expression of p65 RelA and p50 proteins would suggest that pleiotropic signaling induced by CFMs likely involved transcriptional activation of NF-κB. Since CFMs stimulated CARP-1 expression, and CARP-1 was required for HBC growth inhibition by CFM-4 [10], we then determined whether CFM-4 stimulated transcriptional activation of NF-κB and to the extent CARP-1 was required for transcriptional activation of NF-κB by CFM-4. HBC cells were transfected with plasmids pTK/Renilla-Luc and NF-κB-TATA-Luc. Twenty-four hours post-transfections, the cells were either untreated, treated with recombinant TNFα, ADR, or CFM-4 and the cell lysates utilized for determination of luciferase activities as detailed in methods. As shown in figure 6F, each of the agents caused robust stimulation of NF-κB-TATA-driven luciferase activities when compared with the luciferase activities noted in the untreated control cells. Interestingly, knock-down of CARP-1 resulted in significantly reduced NF-κB-TATA-driven luciferase activities in cells that were treated with TNFα, ADR, or CFM-4 (Figure 6F). Our findings therefore are consistent with the known transcriptional activation of NF-κB by TNFα, as well as previously proposed transcriptional activation of NF-κB by ADR. Here we demonstrate for the first time that CFM-4 treatments also cause transcriptional activation of NF-κB, and that CARP-1 expression was necessary for NF-κB activation by TNFα, ADR, or CFM-4. It however remains to be clarified whether activation of NF-κB signaling in the presence of CFM-4 or -5 in NB and HBC cells contributes to apoptosis or serves to promote survival of a small fraction of cells that may eventually emerge from the stressful conditions of treatments with CFMs.

### CFMs target Inhibitor of Apoptosis Proteins (IAPs) in NB cells

MicroRNAs (miRNAs; miRs) are small (∼22 nucleotide in length), intracellular, non-coding RNAs that play important roles in various biological processes. MicroRNAs regulate gene expression by targeting either 3′ or 5′-untranslated regions (UTR) of mRNAs to inhibit translation and thus cause down-regulation of protein expression [31]–[33]. Some of the earlier studies revealed that deregulation of certain miRNAs, often referred to as onco-miRs, contribute to malignancies including lung, breast and prostate cancer [34]–[36]. For example, miR-16 alteration is a key factor in the development of human Chronic Lymphocytic Leukemia (CLL) [37], whereas a decreased expression of miR-*let7* was reported to be associated with progression of lung cancer [38]. To further elucidate NB cell growth inhibitory signaling by CFMs, we investigated whether and to the extent NB inhibitory effects of CFMs involved altered expression of cellular miRs. As a first step, we treated SK-N-SH NB, MDA-MB-468 HBC, and H2373 MPM cells with CFM-4 as detailed in methods. Total RNAs from untreated and treated cells were prepared, and subjected to a high-through-put miRNA profiling. The data showing expression of various miRs in control (untreated) and CFM-4-treated cells are presented as tables S1-S3. A subset of miRs that were regulated by CFM-4 in NB cells is presented in table 1.

**Table 1:** List of select miRs that were differentially regulated by CFM-4 in SK-N-SH NB cells.

ProbeID	Annotation	Average Hy3	SK-N-SH ctrl	SK-N-SH CFM-4; 20 µM; 24 h	logFC
148085	hsa-miR-3687	9,372	8,512	11,031	2,519
145976	hsa-miR-663b	6,803	5,984	8,353	2,369
169159	hsa-miR-4521	7,015	7,807	5,623	−2,183
**146165**	**hsa-miR-1973**	**9,593**	**9,000**	**10,908**	**1,908**
169239	hsa-miR-4732-5p	6,930	6,441	8,243	1,802
46808	hsa-miR-4485	6,581	5,855	7,656	1,801
**168568**	**hsa-miR-1290**	**7,720**	**7,295**	**8,968**	**1,673**
10977	hsa-miR-183-5p	7,104	7,703	6,064	−1,639
168765	hsa-miR-4448	5,806	6,378	4,831	−1,547
168917	hsa-miR-4511	5,687	5,676	7,206	1,530
168672	hsa-miR-1587	7,375	7,099	8,560	1,461
168640	hsa-miR-4475	8,666	8,578	10,029	1,451
169312	hsa-miR-548an	7,061	7,981	6,565	−1,416
147942	hsa-miR-4268	9,770	9,367	10,714	1,347
169034	hsa-miR-642b-5p	10,166	9,732	11,072	1,340
**42581**	**hsa-miR-513a-5p**	**9,240**	**9,162**	**10,475**	**1,313**
168893	hsa-miR-4505	7,743	7,641	8,934	1,293
168572	hsa-miR-4507	6,341	6,067	7,338	1,271
42965	hsa-miR-424-5p	9,422	9,636	8,421	−1,216
145768	hsa-miR-665	7,529	6,867	8,067	1,199
169375	hsa-miR-660-3p	10,651	10,265	11,463	1,198
169326	hsa-miR-451b	8,167	8,624	7,429	−1,195
169381	hsa-miR-4421	8,968	8,648	9,837	1,189
46258	hsa-miR-1184	7,232	8,143	6,971	−1,172
14285	hsa-miR-487b-3p	7,988	8,159	6,990	−1,169
169137	hsa-miR-4524b-5p	6,676	7,001	5,860	−1,142
17888	hsa-let-7a-3p	6,729	7,286	6,153	−1,133
46944	hsa-miR-1297	8,184	8,495	7,380	−1,114
11040	hsa-miR-29b-3p	8,902	9,110	8,002	−1,108
17377	hsa-miR-600	8,291	8,719	7,624	−1,096

Please note that the miRs that were upregulated in CFM-4-treated NB, MPM, and HBC cells are indicated in bold and underlined.

**Figure 7:**
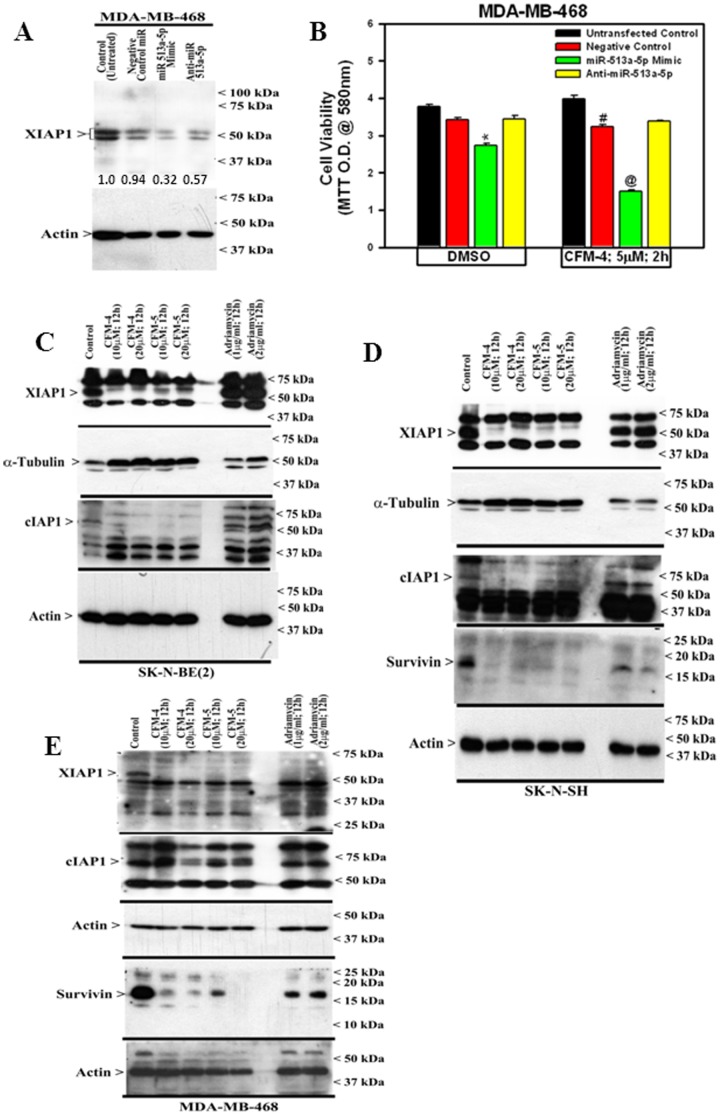
CFM-4 inhibits cell growth in part by inducing a novel miR-513a-5p and suppressing Survivin family of proteins. (A) MDA-MB-468 HBC cells were either untransfected (Control) or transfected with various miRs as noted. Levels of XIAP1 and actin proteins were determined by Western blotting. The signals for the XIAP1 and actin proteins were quantified by densitometry, and the numbers below XIAP1 blot indicate levels of XIAP1 protein in each lane following normalization of the signals with actin levels. For the sake of comparison, the signal intensity for XIAP1 in control (Untreated) lane was assigned an arbitrary value of 1. (B) HBC cells were either untransfected or transfected with various miRs as in A, and subsequently treated with vehicle (DMSO) or indicated time and dose of CFM-4. Determination of viable/live cells was carried out by MTT assay. The data in the histogram represents means of two independent experiments; bars, S.E. *, @, and #, p = <0.03 relative to respective untreated controls. NB or HBC cells were either untreated (Control) or treated with indicated agents for noted time and dose, and levels of XIAP1, cIAP1, survivin, α-tubulin, and actin proteins (C, E) were determined by Western blotting essentially as in figure 2.

Further analysis of the miR profiling data revealed that miRs 513a-5p, 1290, and 1973 were up regulated significantly in HBC, NB, and MPM cells that were treated with CFM-4. The miR-513a-5p was recently found to mediate TNF-α and LPS induced apoptosis via down-regulation of X-linked inhibitor of apoptosis protein (XIAP) in endothelial cells [39]. Inhibitor of apoptosis proteins (IAPs) is family of endogenous inhibitors of caspases and comprise of XIAP, cIAP1, 2, and survivin proteins. IAPs directly bind with and interfere with caspases-3, -7 and -9 to block apoptosis [40], [41]. Survivin is involved in mitosis regulation while its increased expression has been implicated in poor prognosis of NB [40], [41]. We next determined whether growth inhibitory signaling by CFM-4 involved increased expression of miR-513a-5p, and consequent down-regulation of XIAP1. For this purpose, we ablated miR-513a-5p by transfecting anti-miR513a-5p in MDA-MB-468 HBC cells. Negative control miR as well as miR-513a-5p mimic were also transfected as additional controls. WB analysis of the cell lysates revealed that although the transfected cells overall had reduced levels of XIAP1 when compared to the untrasfected control, the presence of miR-513a-5p mimic caused significant reduction in XIAP1 levels in comparison with the XIAP1 levels in the cells that were transfected with negative control miR or anti-miR 513a-5p (Figure 7A). Next, the cells were similarly transfected as in figure 7A, followed by their treatment with 5 µM CFM-4 for 2 h. Although transfection of miR-513a-5p mimic caused a moderate but significant loss of cell viability, CFM-4 treatment of miR-513a-5p-transfected cells resulted in further and significant loss of cell viability (Figure 7B). Consistent with the profiling data where CFM-4 treatments caused elevated levels of miR-513a-5p, transfection of miR-513a-5p mimic not only inhibited cell growth, its presence further enhanced CFM-4 inhibition of MDA-MB-468 cells growth. These data suggest that CFM-4 suppressed growth of MDA-MB-468 cells in part by up-regulating miR-513a-5p, which in turn, targets cell survival regulating XIAP1 protein.

Given that miR-513a-5p targets and down regulates XIAP1 [39], we further clarified whether treatments of NB and HBC cells with CFM-4 or -5 resulted in loss of XIAP family of proteins. Cells were untreated, treated with CFM-4, CFM-5, or ADR, and expression levels of XIAP family of proteins were determined by WB analysis. As expected, CFM-4 or -5 treatments caused depletion of XIAP1, cIAP1 and survivin proteins in both the NB cells (Figure 7C, D). ADR treatments, on the other hand, failed to diminish expression of XIAP1 and cIAP1 proteins in both the NB cells albeit survivin expression was reduced in ADR-treated SK-N-SH cells (Figure 7C, D). Consistent with targeting of XIAP family of proteins by CFMs in NB cells, treatments with CFM-4 or CFM-5 also resulted in reduced expression of XIAP1 and cIAP1 proteins in HBC cells (Figure 7E). ADR treatments although abolished XIAP1 expression but failed to diminish cIAP levels in HBC cells (Figure 7E). Our miR expression analysis together with WB data strongly suggests that CFMs inhibit NB and HBC cell growth in part by targeting XIAP family of proteins.

### CFMs suppress NB cell migration, colony formation and matrix invasion

**Figure 8:**
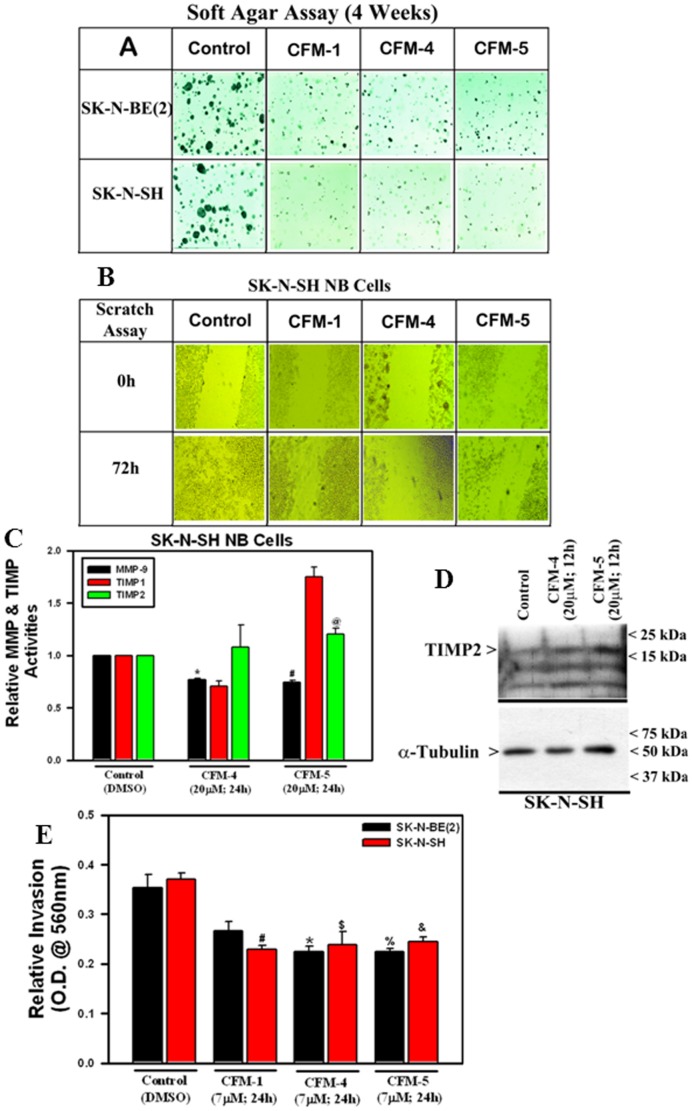
CFMs inhibit NB Cell Growth in Soft Agar, invasion and MMP activities. NB cells were either untreated (Control) or treated with indicated CFMs as in methods, and were subjected to the soft-agar assay (A) or scratch assays (indicated as wound healing assay; panel B). The number if colonies of cells in panel A or cells growth in the scratch assay were recorded by photography as described in Methods. Representative photomicrographs of untreated and CFM-treated NB cells are shown. (C) The SK-N-SH cells were either untreated [Control (DMSO)] or treated with CFM-4 or CFM-5 for noted dose and time. Cell lysates were analyzed for activities of various MMPs and TIMPs as detailed in Methods. The data in the histogram represents means of the activities of the noted MMPs and TIMPs from two independent experiments; bars, S.E. (*, #, and @, p = <0.05 relative to respective MMP and TIMP activities in Control cells). (D) The SK-N-SH were either untreated (Control) or treated with indicated time and dose of respective CFMs. Levels of TIMP2 and α-tubulin proteins were determined by Western blotting essentially as in figure 2. (E) NB cells were separately seeded in chambers with matrigel-coated membranes, and treated with buffer Control (DMSO) or with 7 µM dose of respective CFMs as noted in Methods. Live cells migrating across the matrigel-coated membranes were dissociated, and quantitated by an MTT-based assay. The columns in histogram represent MTT OD of the CFM-treated NB cells relative to untreated controls. (*#, $, %, and &, p = <0.02 relative to buffer-treated, Control cells.

Further, we determined whether CFMs inhibit biological properties of NB cells such as migration, colony formation and invasion, and the molecular mechanisms involved. Exposure of CFM-1, -4, or -5 caused significant reduction in size and number of colonies formed by SK-N-SH and SK-N-BE(2) cells in soft agar as well as prevented SK-N-SH cells from growing in the areas of wound created by a scratch when compared with their untreated counterparts (Figure 8A, B). Our earlier studies have revealed that CFMs caused down regulation of various matrix metalloproteinases (MMPs) in different cancer cell types [14], [15]. Since MMPs and their cognate inhibitors, often referred to as tissue inhibitors of metalloproteinases (TIMPs) are known for their roles in the processes of tissue remodeling, cancer cell invasion and metastasis, and given that CFMs inhibit NB cell migration and colony formation, we next investigated whether CFMs also modulated activities of MMPs and/or TIMPs in NB cells. To test this possibility, we performed antibody-based array analysis to determine the activation of various MMPs and TIMPs in NB cells following their treatments with CFM-4 or -5 as detailed in methods. Although, both CFM-4 and CFM-5 attenuated activities of MMP-1, -8, -9 in MPM cells [14], and MMP-1, -2, -9, -10 in MB cells [15], a moderate but significant loss of only MMP-9 activity was noted in SK-N-SH NB cells that were treated with CFM-4 or CFM-5 (Figure 8C). In addition to MMP-9 inhibition, we also observed moderate albeit significant activation of TIMP2 in SK-N-SH cells that were treated with CFM-4 or CFM-5 (Figure 8C). CFM-5 but not CFM-4 also caused robust activation of TIMP1 in NB cells (Figure 8C). Our WB analysis further revealed an appreciable increase in TIMP2 levels in SK-N-SH cells when exposed to CFM-4 or CFM-5 when compared with their untreated counterpart (Figure 8D). We next examined whether attenuation of MMP-9 and stimulation of TIMP-1 & -2 activities in CFM-treated SK-N-SH cells interfered with invasive properties of NB cells. To test this possibility, we determined the extent to which CFMs blocked the ability of NB cells to invade through matrigel-coated membranes as detailed in methods. As expected, treatments of NB cells with CFM-4 or CFM-5 caused significantly reduced number of cells that were able to invade through the matigel-coated membranes (Figure 8E). Of note here is that although CFM-1 treatments elicited a moderate loss of viability of different NB cells (see figure 1), it was nonetheless effective in blocking NB cell migration, growth in soft agar, and invasion across the matrigel-coated membranes (Figure 8A, B, E). These data together with our earlier studies with MPM and MB models strongly support our hypothesis that CFMs, in particular CFM-4, interfere with NB cell invasion and metastasis signaling in part by targeting MMP-9 activation.

## Discussion

Our previous studies revealed that CFMs are a novel class of compounds that suppress growth of diverse types of cancer cells in part by inducing apoptosis [10], [14], [15]. Here, we highlighted the anti-NB properties of CFMs. CFMs, particularly CFM-4, -5, inhibited NB cell growth by causing G2M cell cycle arrest and loss of mitotic cyclin B1. CFMs stimulated CARP-1 expression and activation of stress-activated p38/JNK and NF-κB signaling pathways. Additionally, our present study revealed that CFMs induced growth inhibition of NB cells that involved down regulation of oncogenic MYCN and c-Myc proteins, while causing up-regulation of miR-513a-5p, which targets and abrogates XIAP-1 expression. In addition to XIAP-1, CFMs also induced loss of cell survival-associated c-IAP1 and survivin proteins.

Previous evidence have shown that MYCN amplification is one of the critical aspects in tumor progression and poor prognosis in NB, and MYCN is often considered an attractive target for therapeutic intervention strategies in this malignancy [42]–[45]. In addition to anti-proliferative properties, CFMs induced loss of MYCN in both SK-N-SH and SK-N-BE(2) cells that have single copy of MYCN and amplification of MYCN, respectively. Since CFMs stimulate CARP-1 expression, and in light of our previous studies demonstrating increased CARP-1-dependent apoptosis in HBC cells expressing reduced levels of c-Myc [6], it is likely that CARP-1 is involved in targeting of MYCN by CFMs in NB cells. Moreover, our current studies revealed that CFMs also targeted c-Myc expression in SK-N-SH NB cells, and the facts that both MYCN and c-Myc belong to Myc transcription family and are well known for their roles in tumorigenesis, our studies highlight ability of CFMs to target Myc family of onco-proteins to transduce their growth inhibitory effects.

We have previously noted a biphasic regulation of NF-κB signaling in MB and MPM cells that were treated with CFM-4 [14], [15]. Consistent with these observations, our current studies revealed that the CFMs also caused a biphasic regulation of NF-κB in NB cells. Although, the CFMs seemed to generally inhibit the canonical pathway of NF-κB activation as evidenced by increased expression of ABINs, expression of IκBα and IκBβ proteins were nonetheless down-regulated in CFM-treated NB cells. This loss of IκBs in CFM-treated cells would be expected to result in nuclear translocation and activation of transcriptional signaling by NF-κB [46]. Indeed our data not only demonstrate that CFM-4 exposure caused a robust transcriptional activation of NF-κB in HBC cells, we show for the first time that CARP-1/CCAR1 expression was necessary for transcriptional activation of NF-κB by TNF-α, ADR, or CFM-4 compounds. Whether and to the extent the NF-κB activation by these agents promoted transcription of cell survival and proliferation regulating genes or was involved in stimulation of apoptosis is not known albeit a number of recent studies have proposed pro-apoptotic functions of NF-κB signaling [47]–[49]. Of note is the fact that although NF-κB regulates transcription of many survivin family proteins including XIAP1, CFMs nonetheless promoted loss of these proteins in NB and HBC cells. It is unclear whether the diminished expression of the survivin family of proteins in the presence of CFMs involves NF-κB-dependent transcriptional mechanisms or the activation of NF-κB occurs to support survival of the cells that are able to eventually overcome the stress and damaging effects of CFMs.

Micro RNAs (miRNAs) are endogenous, single stranded, small non-coding RNA molecules that regulate gene expression at transcriptional and post-transcriptional levels, and are implicated in various biological processes [50]–[52]. Recent studies demonstrated that deregulation of miRNAs contributed to various malignancies. For example, miR15 and miR16 deletion was found in more than 65% of Chronic Lymphocytic Leukemia (CLL) [33], [52]. Consistent with a previous study demonstrating XIAP targeting by miR-513a-5p during TNF-α and LPS-induced apoptosis signaling in endothelial cells [39], our miRNA profiling studies revealed upregulation of miR-513a-5p in CFM-4-treated NB, HBC, and MPM cells. Although, treatments with either CFM-4 or -5 resulted in diminished expression of XIAP1 in NB and HBC cells, CFM-4-treatment of miR-513a-5p mimic expressing HBC cells further enhanced their growth inhibition when compared with their vehicle-treated counterparts. The expression of anti-miR-513a-5p on the other hand interfered with HBC cell growth inhibition by CFM-4, suggesting that miR-513a-5p signaling was likely involved in CFM-4 targeting of XIAP and consequent growth inhibition of HBC and NB cells. Whether similar post-transcriptional targeting of other survivin family of proteins by additional miRs occurs as well as NF-κB signaling stimulates transcription of survivin family of proteins in cells exposed to CFMs, and to the extent the post transcriptional targeting of such mRNAs by miRs is greater than their transcriptional up-regulation by NF-κB to result in overall decreased expression of the survivin family of proteins in the presence of CFMs remain to be clarified.

In addition to miR-513a-5p, the miR profiling also revealed miRs 1290 and 1973 that were also elevated in NB, HBC and MPM cells that were treated with CFM-4 (Table 1). Although a survey of the miR database indicated an array of putative mRNA targets of these miRs, whether and to the extent the miR-1973 functions as a tumor suppressor or onco-mir remain to be determined. In regard to miR-1290, recent reports have revealed that its expression and its potential targets were associated with characteristics of estrogen receptor (ER)α-positive breast cancers while its elevated serum levels were found to be associated with patients with low-stage pancreatic cancers [53], [54]. Interestingly, expression of miR-1290 was also found to favor mitotic exit and differentiation processes of human neuronal progenitors [55]. In this study, upregulation of miR-1290 was noted in SH-SY5Y NB cells that were induced to differentiate into neurons, while ectopic expression of miR-1290 in undifferentiated SH-SY5Y NB cells resulted in a higher number of cells in the G0/G1 phase of cell cycle. Expression of miR-1290 in the undifferentiated neuronal progenitor cells resulted in increase in cyclin-dependent kinase inhibitor (CDKI) p27^kip1^ and a decrease in the levels of proliferating cell nuclear antigen (PCNA). The fact that miR-1290 levels were elevated in ERα-negative MDA-MB-468 HBC cells that were treated with CFM-4 (Table 1) and CFM-4 exposure also resulted in inhibition of ERα-positive MCF-7 cells [10], further studies will be necessary to clarify whether and to the extent elevated expression of miR-1290 contributes to growth inhibition of the NB and HBC cells in the presence of CFM-4.

In conclusion, the studies presented here demonstrate that CFMs suppressed growth of NB cells. CFMs targeted cell survival and growth by down-regulating oncogenes of the Myc family, as well as a number of survivin family of proteins. The robust activation of pro-apoptotic p38 and JNK SAPKs likely served to potentiate NB inhibitory effects of CFMs. CFMs also inhibited MMP-9 activation while stimulating expression of MMP inhibitor TIMP2. MMP-9 was induced in ERK dependent signaling to promote invasion in colorectal cancer [56], and TIMP2 was noted as a transcriptional signature in inhibiting tumorigenesis and metastasis of lung cancer cells [57]. Thus attenuation of MMP-9 together with activation of TIMP2 likely contributed to inhibition of biological properties of migration and invasion of NB cells in the presence of CFMs.

## Supporting Information

Table S1 List of CFM-4-regulated miRs in SK-N-SH NB cells.
(XLSX)

Table S2 List of CFM-4-regulated miRs in H2373 MPM cells.
(XLSX)

Table S3 List of CFM-4-regulated miRs in MDA-MB-468 HBC cells.
(XLSX)
